# Coming together for something good: recommendations from a scoping review for dissemination and implementation science to improve indigenous substance use disorder treatment

**DOI:** 10.3389/fpubh.2023.1265122

**Published:** 2023-10-17

**Authors:** Katherine A. Hirchak, Oladunni Oluwoye, Melanie Nadeau, Meenakshi Richardson, Kelsey Bajet, Mariah Brigman, Jalene L. Herron, Alexandra Hernandez-Vallant, Angel Vasquez, Cuong Pham, Karen Anderson Oliver, Paulette Baukol, Kellie Webb, Lorenda Belone, Michael G. McDonell, Kamilla L. Venner, Aimee N. C. Campbell

**Affiliations:** ^1^Department of Community and Behavioral Health, Elson S. Floyd College of Medicine, Washington State University, Spokane, WA, United States; ^2^Department of Indigenous Health, University of North Dakota, Grand Forks, ND, United States; ^3^Department of Human Development, Washington State University, Vancouver, WA, United States; ^4^Center on Alcohol, Substance Use, and Addictions, University of New Mexico, Albuquerque, NM, United States; ^5^Department of Psychology, University of New Mexico, Albuquerque, NM, United States; ^6^Department of Medicine, University of Minnesota, Minneapolis, MN, United States; ^7^KEAO Consulting LLC, Seattle, WA, United States; ^8^NorthStar Node, Hennepin Healthcare Research Institute, Minneapolis, MN, United States; ^9^Eastern Shoshone Recovery Center, Fort Washakie, WY, United States; ^10^Population Health, University of New Mexico, Albuquerque, NM, United States; ^11^Department of Psychiatry, Columbia University Irving Medical Center and New York State Psychiatric Institute, New York, NY, United States

**Keywords:** dissemination and implementation science, indigenous research methods, community-based participatory research, American Indian and Alaska native adults, indigenous, scoping review, cultural centering

## Abstract

**Introduction:**

Dissemination and Implementation (D&I) science is growing among Indigenous communities. Indigenous communities are adapting and implementing evidence-based treatments for substance use disorders (SUD) to fit the needs of their communities. D&I science offers frameworks, models, and theories to increase implementation success, but research is needed to center Indigenous knowledge, enhancing D&I so that it is more applicable within Indigenous contexts. In this scoping review, we examined the current state of D&I science for SUD interventions among Indigenous communities and identified best-practice SUD implementation approaches.

**Methods:**

PubMed and PsycINFO databases were queried for articles written in English, published in the United States, Canada, Australia, and New Zealand. We included key search terms for Indigenous populations and 35 content keywords. We categorized the data using the adapted and extended Reach, Effectiveness, Adoption, Implementation, and Maintenance (RE-AIM) framework that emphasizes equity and sustainability. RE-AIM has also been used as a primary model to consistently identify implementation outcomes.

**Results:**

Twenty articles were identified from the original unduplicated count of over 24,000. Over half the articles discussed processes related to Reach, Adoption, and Implementation. Effectiveness was discussed by 50% of the studies (*n* = 10), with 25% of the articles discussing Maintenance/sustainability (*n* = 4). Findings also highlighted the importance of the application of each RE-AIM domain for meaningful, well-defined community-engaged approaches.

**Conclusion:**

Finding indicated a need to prioritize Indigenous methods to culturally center, re-align and adapt Western treatments and frameworks to increase health equity and improve SUD treatment outcomes. Utility in the use of the modified RE-AIM and the continued modification for Indigenous communities was also noted.

## 1. Introduction

Many Indigenous communities are interested in providing culturally responsive treatments for substance use disorders (SUD) to their communities (1). While alcohol and rates of other substance use varies greatly across Indigenous communities and reasons for these variation are complex (2–5), there is a need to understand how to better integrate culturally appropriate approaches specific to community and Tribal histories, culture, policy, and concepts of well-being and recovery to reduce the negative consequences of substance use more effectively (3–5). Over the last decade, research on evidence-based SUD treatments among Indigenous people has increased (e.g., motivational interviewing, community reinforcement approach, contingency management), which creates an opportunity to assess the strategies used to implement these treatments in community- and Tribal-based clinical settings (1, 6, 7).

In non-Indigenous focused research, dissemination and implementation (D&I) science models, frameworks, and strategies can guide and facilitate successful adoption, implementation, and sustainment of evidence-based practices to enhance participant outcomes. Indigenous D&I research is emerging and growing (8). Given the historical and ongoing harms created by extractive research practices, it is important that strategies related to implementation of SUD treatment use a community-engaged approach to facilitate equitable research partnerships (9) and work towards health equity.

Recent D&I and intervention research among Indigenous communities has commonly used a community-based participatory research (CBPR) framework to address chronic health conditions and health behaviors related to disease prevention [e.g., hypertension, cardiovascular disease, nutrition, substance misuse prevention and treatment (9) and wellness (10–12)]. CBPR has been widely adopted by Indigenous researchers and community research sites because the approach aligns with Indigenous values of centering the knowledge and expertise of the community, and the importance of Tribal sovereignty with applied outcomes that directly support culturally centered community wellbeing and capacity building. CBPR processes facilitate bi-directional learning and power-sharing between communities and researchers in every step of the process by addressing issues of equity, partnership voice and trust (11–14). While CBPR is thought of as an implementation approach, it has only more recently been conceptualized within the D&I context (15, 16).

Although there are numerous D&I frameworks, models, and theories to support program implementation (17), a commonly used framework to guide implementation outcomes is the Reach, Effectiveness, Adoption, Implementation and Maintenance (RE-AIM) Framework (18, 19) which has recently been expanded to include considerations around contextual factors, longer-term sustainability, and equity (20, 21). RE-AIM is comprised of five domains, each of which reference a particular area of evaluation: **R**each refers to the proportion and characteristics of people who are affected by and engaged in the intervention; **E**ffectiveness is how well the intervention works in a given setting; **A**doption is the proportion and representativeness of participating and non-participating providers and settings; **I**mplementation is the extent to which the intervention is delivered as intended (e.g., fidelity); and **M**aintenance (or Sustainability) is the extent to which the intervention becomes part of routine practice, as well as the long-term impact of the intervention.

While RE-AIM has evolved and been used within a range of contexts over the years (19), misconceptions about the framework persist. These assumptions primarily fall within four areas: that RE-AIM is simply for evaluation, the framework privileges quantitative over qualitative data, all dimensions must be weighted the same, and that the Maintenance phase encompasses only 6 months (22). Recently published studies have clarified that when appropriate (22–25), RE-AIM can incorporate qualitative and mixed methods designs, is not restricted to evaluation, and can be applied during dissemination, adoption, planning, and implementation (23). In addition, the framework has been expanded to include greater emphasis on sustainability (e.g., implementation for more than 1 year, integrating internal and external factors influencing implementation success) and health equity (21). For practitioners and researchers, applying RE-AIM during different phases of the project, while also maintaining a health equity and sustainability lens, better supports a multi-level approach that addresses the evolving needs related to capacity, as well as barriers and facilitators that many programs, organizations, and minoritized communities face. For Indigenous communities, the inclusion of a health equity and sustainability lens is particularly relevant for SUD treatment given health disparities in substance use related health outcomes, funding and capacity difficulties faced by Tribal health organizations.

The purpose of this scoping review is to inform future SUD evidence-based implementation research among Indigenous communities. A scoping review was identified by the study team as the appropriate approach because of the nascent nature of the state of the field. We sought to assess the existing evidence, clarify key concepts, and identify potential next steps to advance the science (26). While to our knowledge there are few completed published implementation trials for SUD interventions among Indigenous communities, we sought to inform future research and support practice change by examining a broad range of implementation strategies and processes among the more general Indigenous SUD treatment literature. Additionally, we reviewed whether Indigenous frameworks and worldviews have been centered in this work. Our guiding research question was what implementation processes or strategies are used among Indigenous communities for the uptake of evidence-based substance use treatment. We characterize the relevant research using the RE-AIM domains, contributing to previous work that has applied the framework with a focus on implementation (24, 25).

## 2. Methods

### 2.1. Sources and search protocol

Literature searches and eligibility assessment occurred in June and July of 2021 using Preferred Reporting Items for Systematic reviews and Meta-Analysis guidelines [PRISMA-Equity; search activities were completed by author MR (27)]. The search strategy was conducted within PsycINFO and PubMed/Medline databases. The second step in the search protocol involved all combinations of our population and content keywords. Population keywords included words used to describe and identify Indigenous populations. There were more than 30 content keywords including reference to dissemination and implementation frameworks and approaches. Combinations of each population term and all content keywords were administered via advanced search options with selections made for peer-reviewed articles written in English.

Search keywords were either combined with “AND” or in one plain search phrase (e.g., American Indian and Alaska Native; Māori “AND” dissemination and implementation science; implementation strategies; Explore, Preparation, Implementation and Sustainment; health equity implementation framework; Indigenous Implementation Framework). For a complete list, please refer to the Supplementary materials. The third step in the research strategy was to take the references identified by all combinations of search terms from each database extraction and save them as Research Information Systems (RIS) text files that were then uploaded into COVIDENCE, a systematic review management software (28). Duplicates were identified and removed within the software system.

### 2.2. Study selection

KH (descendent of the Eastern Shoshone Tribe/White, mixed European ancestry), MR (citizen of the Haliwa-Saponi Tribe), and KB (descendant of Filipino ancestry and immigrants) reviewed research articles for eligibility in COVIDENCE based on the title and the abstract. Study eligibility criteria was determined by the research team using knowledge of existing literature (e.g., anticipated state of the science), consideration of limitations of the research team (e.g., language capacity), and specific interest in SUD treatment implementation (29). Inclusion criteria were: (a) peer-reviewed, (b) written in English, (c) conducted with an Indigenous, adult (18 and older) population, (d) comprised of aims and outcomes related to substance misuse; (e) delivering an evidence-based intervention or practice based upon Western scientific criteria (e.g., efficacy or effectiveness randomized controlled trials), and (f) described one or more implementation processes or strategies. Exclusion criteria were: (a) studies completed entirely with non-Indigenous populations, (b) interventions intended for youth (younger than 18 years-old), (c) research that did not include outcomes related to substance use, and (d) research that did not discuss an evidence-based intervention (e.g., efficacy or effectiveness randomized controlled trials) for substance use. There was no exclusion based on publication date.

### 2.3. Data extraction

For articles which met initial eligibility criteria based on title and abstract, a full text review was conducted by KH and MR. Data extraction was completed by KH, MN (Turtle Mountain Band of Chippewa Indians), MR, KB, and MB (Spokane Tribe of Indians). Articles were initially reviewed independently and then underwent a second review by an alternate author to ensure validity and reliability of the extraction. None of the reviewers were individuals who had authored one of the articles. Consensus was determined and resolved by KH, MR, KB and MB. Final data review was carried out by KH and MR.

### 2.4. RE-AIM domain application

Once the final sample of articles was confirmed, the adapted RE-AIM framework was utilized for coding by two independent coders. While none of the studies were explicit D&I outcome studies, we coded the articles based upon what applied to the RE-AIM framework. This included articles that were clinical trials, methods papers, qualitative research, and case studies. Operational definitions based on the RE-AIM framework were identified and developed by the first author along with the review protocol (see Supplementary materials adapted from D’Lima et al. (30) and Shelton et al. (21)). The health equity and long-term sustainability extension codes were embedded within each existing domain. Codes were used to complete the data extraction within COVIDENCE, with final consensus completed in Excel.

For Reach, two questions were coded around the intended audience and who participated, as well as ways to better reach and engage the intended audience. Effectiveness was coded with three items related to whether the intervention was effective, if there had been any unintended consequences, and if intervention effectiveness was assessed over time. Adoption was assessed by three questions at the staff and setting level. This included application of the intervention and by whom, which staff were invited to participate versus excluded, and how staff were supported in delivering the intervention or could have been better supported. Implementation was assessed by six items ranging from efforts to culturally adapt the intervention, fidelity and delivery of the intervention, and costs. Maintenance was reviewed through the lens of sustainability of the intervention beyond 1 year and if multi-level contextual determinants were discussed that might impact sustainment.

## 3. Results

### 3.1. Description of articles

**Figure 1:**
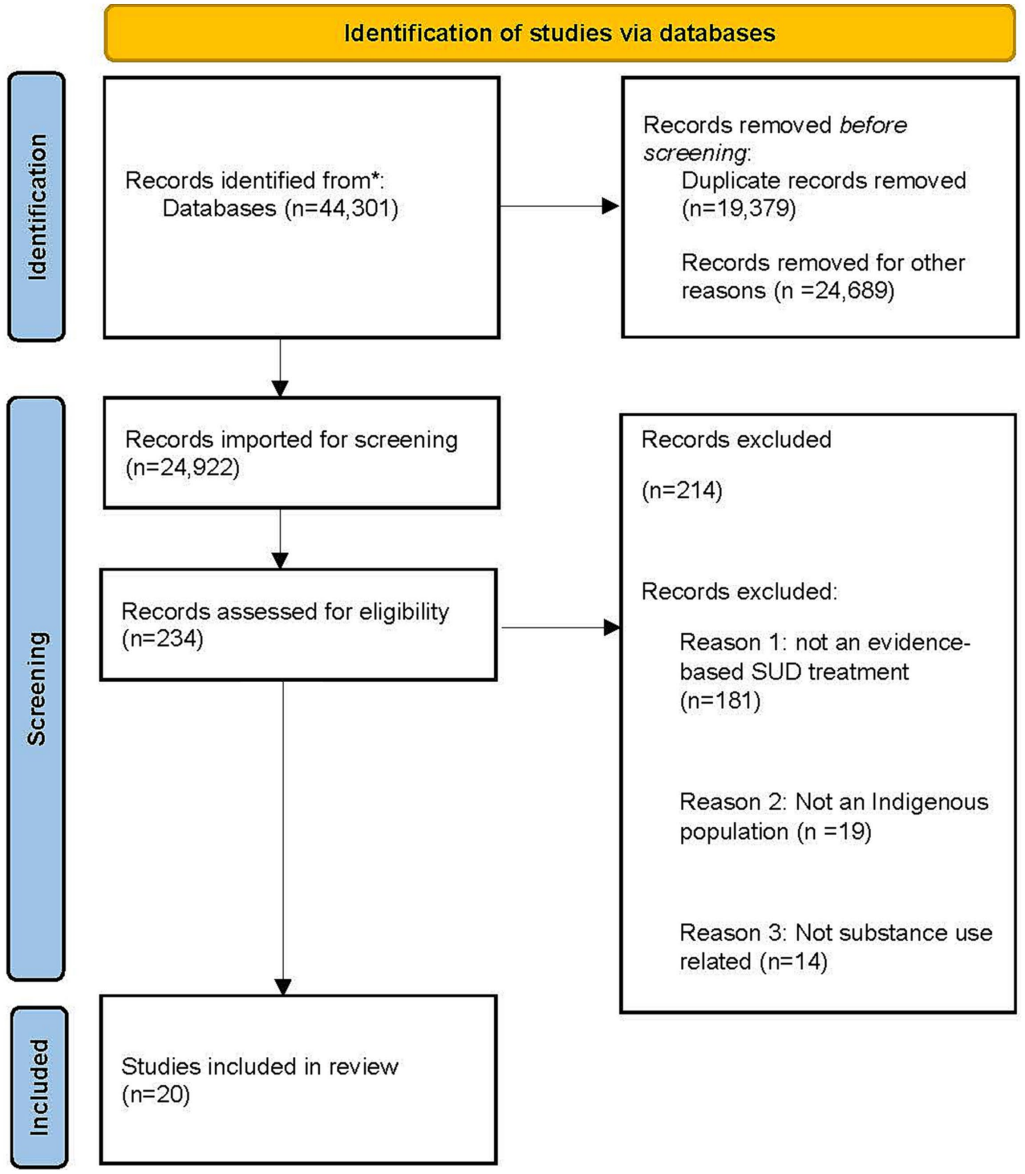
PRISMA flow diagram. Adapted from Page et al. (31).

The initial search produced 44,301 articles. After the removal of 19,379 duplicates, an additional 24,689 were deemed ineligible through title and abstract screening and 234 articles underwent full-text review. After the full text-review, 20 articles were identified for inclusion (see Figure 1). Just over one-third were methods or protocol papers and about half of the studies included a randomization component. Of the studies identified, 65% included AI/AN adults, 20% Aboriginal Australian adults, 10% First Nations adults, and 5% Native Hawaiian adults. The evidence-based SUD programs being implemented (sometimes in combination) included: nicotine replacement therapy (*n* = 8), motivational interviewing (*n* = 5), community reinforcement approach (*n* = 4), medications for opioid use disorder (*n* = 3), contingency management (*n* = 3), community reinforcement approach and family training (*n* = 2), and recovery housing (*n* = 1). Primary substance use outcomes included smoking cessation (*n* = 7), alcohol use (*n* = 6), more than one substance or not specified (*n* = 5), and opioid use (*n* = 3). All the interventions focused on the individual, with a little over half also having a family, community, or organizational component (*n* = 12). Only two of the studies intentionally included implementation strategies and existing dissemination and implementation frameworks, but this was integrated retrospectively (Table 1).

**Table 1:** Frameworks and sample cultural adaptations.

Author	Indigenous community	Evidence based treatment culturally re-centered	Community engaged or CBPR	Western adaptation/implementation frameworks	Indigenous frameworks	Sample cultural re-centering of evidence based treatment
Bar-Zeev (32)	Aboriginal Pregnant Women, Australia	No	No	No	No	Stakeholder and Consumer Aboriginal Advisory Panel Cultural liaison
Burduli (33)	AI Adults, United States	Yes	Yes	No	No	Culturally appropriate rewards Elders and community members led the intervention
Calabria (34)	Aboriginal and Torres Strait Islander Adults, Australia	~	~	No	No	~
Campbell (35)	Urban AI/AN Adults, United States	No	Yes	No	No	No
Daley (36)	Urban AI/AN Adults, United States	Yes	Yes	No	No	Ceremonies, traditional and cultural activities related to tobacco, coping and stress Culturally appropriate rewards
Duvivier (37)	American Indian Adults, United States	Yes	Yes	No	No	Pharmacists developed culturally responsive materials Efforts to reduced stigma and build individual/community trust
Gould (38)	Aboriginal Pregnant Women, Australia	Yes	Yes	No	No	~
Hanson (39)	AI/AN Adult Women, United States	Yes	Yes	No	No	Community members led the intervention Community Advisory Board
Hirchak (40)	AI Adults, United States	Yes	Yes	Interactive Systems Framework ADAPT-ITT	No	Focus groups Speakers of the Native language led sessions
Hirchak (41)	AI/AN Adults, United States	Yes	Yes	Quality Implementation Checklist	No	Community Advisory Board Community members led the intervention
Jason (42)	Snohomish Tribe, United States	Yes	Yes	No	No	Residents tailored processes to meet their needs (e.g., talking circles, Indigenous artwork)
Kiepek (43)	First Nations, Canada	Yes	Yes	No	No	Foods and medicines for a traditional diet provided Elders-in-residence to lead cultural activities and ceremonies
Landry (44)	Elsipogtog First Nation (MiÍûåkmaq First Nations Band), Canada	Yes	Yes	No	No	~
Marley (45)	Miriuwung and other Aboriginal and Torres Strait Islander Adults, Australia	~	Yes (PAR^a^)	No	No	Community members led the intervention Historical and present factors impacting communities specifically described
McDonell (46)	AI/AN Adults, United States	Yes	Yes	No	No	Focus groups Culturally appropriate rewards Community members led the intervention
Orr (47)	Rural AI/AN Adults, United States	Yes	Yes	No	No	Focus groups Materials adapted (e.g., culturally relevant text messages)
Patten (48)	Yup’ik, Cup’ik or Athabascan, United States	Yes	Yes	Cultural Variance Framework Surface and Deep Structure Framework	No	Counseling included an emphasis on positive cultural and community activities to cope with tobacco withdrawal Community members led the intervention Integrated traditional cultural practices, values and teachings (e.g., Yup’ik ways of being healthy)
Santos (49)	Native Hawaiian Adults, United States	Yes	Yes	No	No	Materials were culturally responsive and included values of family, solidarity, and motivators for behavior change Well respected community member and champions integrated for cultural relevance
Venner (50)	AI Adults, United States	Yes	Yes	No	No	~
Venner (1)	AI Adults, United States	Yes	Yes	No	No	Speakers of the Native language led sessions Traditional introductions Culturally adapted and validated measures

~Unclear.

a Participatory Action Research.

### 3.2. Characterizing studies through the RE-AIM domains

**Figure 2:**
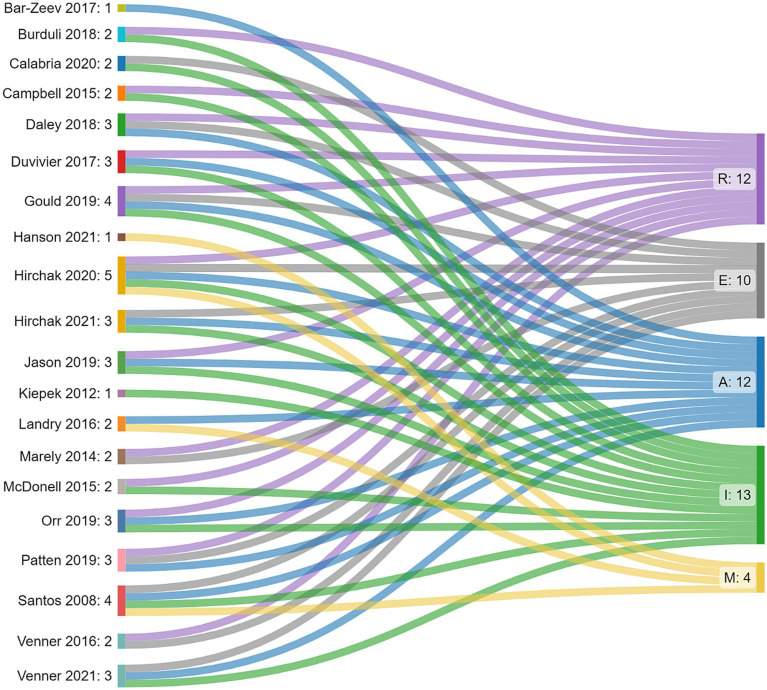
Distribution and frequency of RE-AIM domains assessed and discussed within each study.

Reach, Adoption, and Implementation were discussed by 60% of the studies. This was followed by Effectiveness (50% *n* = 10), with five studies discussing Maintenance/sustainability (25%, Table 2). Frequency and distribution of each of the domains coded in the literature are also presented (Figure 2). Below we provide descriptions of how the RE-AIM domain was characterized and include examples from the literature that highlight each domain. Definitions of domains and abbreviated summaries of examples can be found in Table 3.

**Table 2:** Summary of RE-AIM domains included in each study.

Author	EBP	Study design	Reach	Effectiveness	Adoption	Implementation	Maintenance
Bar-Zeev (32)	NRT	Methods/ Protocol Paper (SWRCT)^a^	No	No	Yes	No	No
Burduli (33)	CM	Methods/ Protocol Paper (RCT)^b^	Yes	No	No	Yes	No
Calabria (34)	CRAFT	Quasi-Experimental	No	Yes	No	Yes	No
Campbell (35)	TES (CRA)	Mixed-Methods	Yes	No	No	Yes	No
Daley (36)	NRT/ MI	Single Arm Clinical Trial	Yes	Yes	Yes	No	No
Duvivier (37)	MOUD	Descriptive	Yes	No	Yes	Yes	No
Gould (38)	NRT	SWRCT^a^	Yes	Yes	Yes	Yes	No
Hanson (39)	MI/ CDC CHOICES	Methods/ Protocol Paper (RCT)^b^	No	No	No	No	Yes
Hirchak (40)	MI/ CRA/ CRAFT	RCT (Case Study)^b^	Yes	Yes	Yes	Yes	Yes
Hirchak (41)	CM	RCT (Case Study)^b^	No	Yes	Yes	Yes	No
Jason (42)	Oxford House	Case Study	Yes	No	Yes	Yes	No
Kiepek (43)	MOUD	Case Study	No	No	No	Yes	No
Landry (44)	MOUD	Qualitative	No	No	Yes	No	Yes
Marley (45)	NRT	Qualitative	Yes	Yes	No	No	No
McDonell (46)	CM	Methods/ Protocol Paper (RCT)^b^	Yes	No	No	Yes	No
Orr (47)	NRT/ STOMP	Methods/ Protocol Paper (RCT)^b^	Yes	No	Yes	Yes	No
Patten (48)	NRT	Methods/ Protocol Paper (CRCT)^c^	Yes	Yes	Yes	No	No
Santos (49)	NRT	Case Study	No	Yes	Yes	Yes	Yes
Venner (50)	MI/ CRA	Quasi-Experimental	Yes	Yes	No	No	No
Venner (1)	MI/ CRA	RCT^b^	No	Yes	Yes	Yes	No

Adapted from D’Lima et al. (30).

a Stepped wedge randomized controlled trial.

b Randomized controlled trial.

c Cluster randomized controlled trial.

**Table 3:** RE-AIM domains and sample process.

**Domain**	**Definition**
**Reach**	% of individuals excluded, characteristics of those excluded, number or proportion of representativeness of individuals who participated, how to better engage the intended audience
**Examples from the literature**	
- 142 participants were screened for a alcohol and illicit drug contingency management intervention, but only 114 met inclusion criteria [i.e., self-reported AI adult 18+; seeking alcohol misuse or dependence and drug misuse or dependence treatment; a Diagnostic and Statistical Manual, fourth edition diagnosis of current alcohol dependence; current drug misuse defined as drug use in the last 30 days; ability to provide informed consent, read and speak English; Burduli et al. (33)] - The researchers described having a Community Advisory Board to guide the intervention and completed focus groups to culturally adapt and gain community feedback on study materials and processes to better engage the intended audience (46)	
**Effectiveness**	Was the intervention effective, for whom was it effective
**Examples from the literature**	
- In a tobacco cessation program specifically adapted for American Indian adults, after accounting for attrition, the overall quit rate at the end of the 3-month intervention period was about 41% and at 6 months post-baseline the final quit rate was approximately 22% [*p* = 0.002; Daley et al. (36)] -AI/AN participants who received a chance to draw for prizes when they submitted an alcohol-negative urine sample were 70% more likely to be alcohol abstinent compared to those in the control group (41)	
**Adoption**	By whom and where was the Evidence Based Treatment implemented, who was invited or excluded from implementation, intervention consistently applied (fidelity)
**Examples from the literature**	
- A tobacco cessation program among pregnant women in Alaska had the intention to hire a “Native Sister” from each participating village, but that this had not been feasible. One “Native Sister” was hired out of the Bethel, Alaska area to deliver the intervention at all the villages that did not already have one. Ultimately, the Bethel area had the best recruitment numbers because the prenatal visits were also located there (48) -MICRA had many supports related to counselor fidelity and intervention delivery. Experts in MI and CRA delivered the training and on-going technical assistance. Counselors were then also able to deliver the intervention in their Native language to further strengthen intervention adoption (1, 50)	
**Implementation**	Consistency of implementation strategies, costs of delivery of Evidence Based Treatment discussed, implementation strategies adapted/culturally adapted
**Examples from the literature**	
- In a self-paced educational module curriculum of TES, implementation strategies were consistent and included a multi-component approach of ongoing consultations from AI/AN clinical administrators, staff, and researchers on the development of the proposal, training, recruitment, and assessments. Collaborators made contributions to the interpretation of the data as well as the development of dissemination materials (35) - In a text messaging for tobacco cessation intervention, eight focus groups were conducted at four Tribal colleges in Montana, to support the cultural adaptation of the text messages. A total of 55 AI/AN Tribal college students who were currently smoking or were previous smokers participated across the 8 focus groups. The original messages designed for Māori young adults, and were reviewed by 2 AI research team members, all Māori specific messages and references were removed and those that were consistent to AI/AN cultures were retained for focus group review. This process yielded 104 culturally adapted text messages, of those 30 were newly developed by the focus groups (47)	
**Maintenance**	Implementation strategies discussed to sustain the program long-term or beyond 1 year after implementation
**Examples from the literature**	
- An intervention that addresses risk of alcohol-exposed pregnancies, discussed their plans for an economic evaluation that would be completed at the end of the 5-year study to assess the cost-savings related to the intervention and among participants in reducing alcohol related harms, and to assist future work in program sustainability (39)	

Adapted from Glasgow et al. (19) and Shelton et al. (21).

### 3.3. Reach

Sixty percent of the studies reported Reach [*n* = 12; (33, 35–38, 40, 42, 45–48, 50)] with information mainly focused on inclusion and exclusion criteria for participants along with recruitment strategies (33, 35, 36, 40, 42, 45, 46, 48, 50). For example, Campbell and colleagues (35) noted that they recruited 58.8% (*N* = 40) out of the 68 clients that were eligible to participate in their study. In another study, the involvement of treatment providers from the community was also critical to Reach, with one study describing their recruitment efforts as including clinician referrals, radio newspaper, and digital (i.e., Facebook) ads, outreach at community events, and word-of-mouth (33). Reach was expanded in a pharmacist-led initiative through the Indian Health Service to increase access to medications for opioid use disorder [MOUD; (33)]. This was achieved by increasing the number of individuals initiated onto medications, poisoning awareness and procurement of naloxone kits that were distributed nationally. Other strategies around Reach included naloxone/drug take-back initiatives and engagement through culturally responsive educational tools (37).

Within the Reach domain, though not always referencing CBPR specifically, articles highlighted the importance of community buy-in and engaging collaborators on the project that were a part of the community and had knowledge of cultural protocols [e.g., how to appropriately greet an Elder; (50)]. Another common implementation strategy across studies was convening an advisory board (e.g., Community Advisory Board) or collaborative board to provide oversight on research activities, assist with recruitment and facilitate connections across organizations in the community. In one study, for example, the Community Advisory Board assisted in developing the culturally appropriate intervention title and how to brand the project in each community, identified and adapted measures, and guided focus groups to increase cultural acceptability of the intervention (40).

Trust was also emphasized in a few studies as another reason Reach was successful (36, 37). A trusting relationship between the participants and the research team was key. In these studies, it was argued that participants reported that they participated because they knew that the research team would manage their data with care, which speaks to data sovereignty and privacy. A unique aspect of Reach and trust was illustrated by a study completed in Australia (45). The authors described how “jealousy” played a significant role in recruitment. As reported by the authors, the spouses of participants were concerned that participation in research would provide opportunities for romantic infidelity. Research staff responded by developing strategies to make the intervention more welcoming and inclusive by providing opportunities to non-participating spouses. While specific to this community, this study demonstrates the importance of an engaged and flexible staff that can identify barriers and then develop solutions.

The need for flexibility in participant engagement strategies, and the ability to pivot based on community-generated solutions for health equity in substance use accessibility and capacity was, in fact, described across studies. For example, Orr and colleagues (47) initially intended to recruit AI/AN college students for their tobacco cessation program. However, recruitment efforts led to only 9 college students enrolling. The researchers then turned their recruitment strategy to Quitlines in states that had higher AI/AN rural populations (e.g., Alaska, New Mexico) and were able to complete their recruitment efforts. Another study identified recruitment challenges due to staff not having the time to recruit at the site, a short recruitment window, and eligibility criteria that were too stringent (38). One study team discussed needing to make changes to recruitment strategies when participants were screened eligible but declined to participate. Of note, the reasons for declining varied but were both practical and directly related to the research fit in the community. For example, participants reported not being comfortable with randomization, lack of interest in research, a preference for another type of treatment, and study location that was too far away (50).

### 3.4. Effectiveness

Out of the 20 studies reviewed, ten (50%) of them reported outcomes related to intervention effectiveness (1, 34, 36, 38, 40, 41, 45, 48–50). There were several studies where the primary outcome was tobacco cessation [*n* = 7; (32, 36, 38, 45, 47–49)]. In a tobacco cessation program for pregnant Aboriginal women, self-reported 12-week 7-day point-prevalence abstinence was 13.6% (38). In another smoking cessation study integrated across two geographically and culturally distinct Aboriginal primary care settings, no statistically significant difference was observed in quit rates, but the study was also statistically underpowered (45). Although the quit rate varied, up to 43% of patients were tobacco free for 3 months across a healthcare system with five locations serving Native Hawaiian people (49).

Interventions addressing alcohol and other substance use also resulted in favorable outcomes. In a study assessing the effectiveness of Motivational Interviewing and Community Reinforcement Approach among a Tribal community in the Southwest United States, at 8 months, percent days abstinent had increased for both alcohol and cannabis, as well as other substances [excluding tobacco; (47)]. In the primary outcome clinical trial of Motivational Interviewing and Community Reinforcement Approach, participants in both the treatment, and treatment as usual (TAU), improved in percent days abstinent and substance use severity [MICRA PDA =72.63%, TAU = 73.62%; (1)]. At the three-month follow up in another study, Aboriginal participants in the Community Reinforcement Approach intervention had significantly reduced their alcohol use. There were also reductions in frequency of days of alcohol consumed and number of drinks consumed per drinking day based on pre- and post- self-report (34).

### 3.5. Adoption

Sixty percent of the studies described processes and outcomes related to Adoption [*n* = 12; (1, 32, 36–38, 40–42, 44, 47–49)]. At the staff level, authors highlighted strategies, for and barriers to, Adoption. Including enhancements to Adoption focused on the staff members themselves. While some studies had specific inclusion and exclusion criteria for staff (32, 38, 48), Indigenous staff and level of staff buy-in were highlighted as the key to increasing Adoption (37, 41, 44). The duration of the intervention also impacted Adoption. While shorter interventions are generally considered easier to Adopt, some of the studies highlighted that shorter project duration among Indigenous communities actually inhibited long-term Adoption and more time was needed to build relationships (36, 38, 40). Recommendations to increase Adoption included hiring qualified study staff who were trusted community members cross-trained to facilitate coverage (38). Also, the quality of the Tribal-organization and university partnership and the need for relationship-building at all levels was of noted importance, for managing potential distrust of research by the community, as well as to facilitate approval processes from multiple entities (1, 33, 41, 46).

At the organizational level, Adoption was discussed in the context of recruitment success, with higher recruitment translating to better adoption (36, 41, 45). One study focused on tobacco cessation located at a healthcare system with different locations across the Hawaiian Islands. Differences in adoption by site were explained by barriers such as lower level of administrative or clinical support, staff turnover, and lower organizational readiness to adopt a new protocol or service (49). Although the authors initially assumed locations would be similar because they were within the same health system, each of the five locations evidenced variation in community needs, representation on the governing board, use of their medical record system, or attitudes toward medications for tobacco cessation (49).

### 3.6. Implementation

Facilitators of Implementation were described at both the internal (i.e., organizational) and external levels, with 60% of studies touching upon this [*n* = 12; (1, 33–35, 37, 38, 40–43, 46, 47, 49)]. Organizations face an array of internal challenges (e.g., shifting priorities and reduced funding) along with external factors that impact what types of interventions may be supported or can be reimbursed. Authors discussed strategies related to the need for capacity building within and outside of the organization, having champions at multiple levels within (e.g., direct service, supervisor levels) and outside of the organization [e.g., Tribal leadership; (1, 40, 41, 49, 50)] and holding information meetings about the intervention for service providers outside of the organization (i.e., community members and leaders). Within the organization, it was recommended that there be on-going face-to-face communication between research staff and site staff and sharing of information and lessons from Implementation including monthly ongoing technical assistance/facilitation (1, 35, 38, 40, 41, 45).

Modifications to the intervention to enhance acceptability, integration, and implementation included cultural adaptations tailored to each setting. Making cultural adaptations to evidence-based substance use disorder treatment was described as a more holistic approach that sits within the cultural framework of the partnering community. This can take the form of integrating appropriate representation (e.g., art, study staff from the community) knowledge, and Indigenous worldviews into the evidence-based treatment which may also increase engagement and adoption (33, 42, 43, 50) (Table 1). Seventy-five percent of the studies (*n* = 15) mentioned culturally adapting the intervention, but only 15.0% (*n* = 3) described a specific framework guiding the adaptations made. While 90% of the studies mentioned a process of community engagement, only one-third of the studies explicitly stated the use of CBPR. Of those, there was no description or definition of how CBPR was being interpreted. None of the studies explicitly identified an Indigenous framework used as a part of the program implementation or intervention adaptation.

Examples of the commonly described cultural adaptation processes and methods included: focus groups, having members of the community lead the intervention, and culturally adapting materials (e.g., including Indigenous languages or pictures that represent the community). One study was initiated when a Tribal elected official contacted the researchers following the development of a community sober living house to assist with evaluation of feasibility, acceptability, and effectiveness (42). The study was unique in that the residents culturally adapted the model and the implementation processes in real time. The communal nature of the sober living house aligned with Tribal culture, and participants were able to include cultural activities (e.g., sweat lodge, talking circles with the inclusion of sacred objects like feathers, and drumming circles). Further modifications included the house being run by the residents who decided all activities, house rules and procedures. The house was also inclusive of non-Tribal members, so that anyone would have a home if they needed one, reflecting the Tribe’s values of providing social support and resources to those most in need (42).

Although funding is a core aspect of Implementation, it was not frequently discussed. Many of the studies did not directly specify the costs of delivering the evidence-based treatment, and, when funding was discussed, studies highlighted that it had enhanced Implementation efforts. For example, one study described how the total budget was dispersed across partnering communities during the duration of the research project and that this had been one of the major strengths of the collaboration [e.g., $1.8 million budget over 6 years for contingency management; (48)]. The authors of a multi-component intervention for smoking cessation among pregnant Aboriginal women did not list the entire cost of the intervention and delivery but noted that each site received $6,000 to support delivery of the intervention and staff contribution (38). In another study assessing recovery housing, Oxford Houses were argued to be self-sustaining and a lower cost option for post-treatment or recovery maintenance because each resident contributed to the cost of running the home. Professional staff were not employed (Oxford houses are operated by the residents) and residents’ rent typically amounted to about $100 per week (42).

### 3.7. Maintenance/sustainability

Maintenance, or Sustainability, was the least discussed across studies with 20% of the articles including some discussion around this domain [*n* = 4; (39, 40, 44, 49)]. Multi-level factors related to sustainment suggested by a few authors included societal and policy forces (e.g., racism, historical trauma, poverty, and discrimination) but these components were not directly assessed within the studies (1, 41, 44, 45, 50). The studies that did highlight Sustainability also speculated on activities that may support or hinder the long-term delivery of the intervention. Authors also described future plans for program sustainment (39).

One qualitative study that examined a program delivering medications for opioid use disorder emphasized the need for community education and buy-in around harm reduction strategies to effectively sustain program activities. The need for education and training related to these medications was especially important to inform family members about how the medications worked to support treatment and how they were different from other types of opioids and illicit drugs (e.g., some family members were unsupportive of methadone maintenance treatment). Among those interviewed, there was a belief that the long-term impact of the medication for opioid use disorder program had been to increase cleanliness/safety (e.g., reduction in discarded needles) and reduce crime (e.g., burglary, vandalism) in the community. The program had already been on-going for 5 years by the time of publication, so institutionalization of the program was achieved, but the specifics of how this was accomplished were not discussed in detail (44).

## 4. Discussion

In this scoping review, we assessed implementation strategies and processes employed by researchers and Indigenous communities to enhance the uptake and delivery of evidence-based substance use disorder treatments, applying a widely used public health framework, RE-AIM: Reach, Effectiveness, Adoption, Implementation, and Maintenance. Across eligible studies, the most often discussed processes were Implementation, Reach, and Adoption followed by Effectiveness and Maintenance/sustainability. Of the 10 studies that described Effectiveness, all but one intervention led to statistically significant improved substance use outcomes. The present study highlights the need for additional implementation research with Indigenous communities to better understand implementation strategies and outcomes that will support the integration of evidence-based practices to address substance use disorders.

Many of the studies referenced the importance of a community engaged or CBPR approach but few defined or detailed what this meant or how the CBPR methods were applied. Several articles also mentioned having a Community Advisory Board or Research Review Board, which are key principles of CBPR, but how exactly this was developed and deployed remained unclear (for examples see 51, 52). Additionally, most of the studies did not outline the use of specific frameworks, or dissemination and implementation theories and models. Future research should be model-driven and systematically describe the methods used with Indigenous communities to support this emerging knowledge base. Strategies and approaches in D&I research must also be pursued thoughtfully so that an emphasis or privileging of Western worldviews does not hinder the use or development of Indigenous frameworks to braid collaborations [e.g., the Indigenist-Stress Coping Model (53), the Two-Eyed Seeing approach (54) or the He Pikinga Waiora Implementation Framework; (55)]. As noted by McCuistian and Colleagues (56), a need exists for a well-specified model and plan for community engagement and CBPR approaches with minoritized communities. This is likely to support reach and engagement within the community and ensure that the intervention is desired and integrated appropriately for and with the community. Prior evidence also suggests that this may lead to improved intervention effectiveness (57).

A primary challenge to sustainability from the studies assessed in this review was lack of or insufficient funding. Lack of program sustainment creates further issues around trust between researchers and communities (e.g., when programs or research projects are visible in the community and then suddenly disappear when the funding ends). Challenges in program sustainment also inhibit the ability to truly impact health equity (58). Additionally, we found that despite the large sample of articles identified through the initial search, there were few that described implementation research specifically for SUD treatments with Indigenous communities. More work is needed that is culturally congruent or grounded (and specifies the methods for conceptual grounding) to increase both the literature on implementation strategies as well as the literature on evidence-based treatments.

Another component of our analysis was related to health equity and the integration of Indigenous frameworks. Findings indicated that while some of the articles intended to positively impact substance use outcomes, many did not discuss the multi-level determinants necessary for interventions to work [e.g., the social determinants of health or the importance of overall improved quality of life; (57)]. A few authors suggested that societal and policy forces (e.g., racism, historical trauma, poverty, and discrimination) should be considered (1, 41, 44, 45, 50), but it was not explicit as to how the interventions might seek to address these factors. It is important that future SUD treatment research is placed within the larger colonial-settler context so that Indigenous people are not further stigmatized or blamed for the consequences of colonization and to ensure that meaningful systems change occurs.

There were notable strengths of this review and several limitations. Reviewers may not have extracted all relevant data for the specified domain due to lack of clarity or explicit description within the article. However, our analysis of these data included independent assessment from at least two reviewers and consensus building across reviewers to increase scientific rigor. Second, this research was focused on evidence-based treatments and used a Western framework to structure the review. This may have reduced our ability to examine community-centered approaches. While our final sample included 20 articles, the initial unduplicated count was close to 25,000. We believe this was due to the extensive population and content search terms (35+; see Supplementary materials). Future studies may want to include fewer keywords and more focused search terms to summarize any recent literature accordingly, as this research area continues to grow. Although we used the PRISMA-Equity Extension checklist, future research might consider the development of an *a priori* review protocol. Despite this, a strength of this review was the extensive assessment of implementation strategies and processes used by researchers and communities to effectively increase the health and well-being of Indigenous people.

## 5. Conclusion

Among Indigenous communities, the field of D&I is expanding. Research partnerships have led to the implementation of culturally adapted evidence-based treatments for substance use disorders that are more in alignment with culture and holistic conceptualizations of health and well-being. Given the continued growth in the field, we provide 6 broad recommendations from our findings to move the science forward. (1) The need for more SUD interventions among Indigenous communities that also include ways to address or reduce the disproportionate health inequities related to the social determinants of health that contribute to substance use. (2) While some studies addressed factors related to Implementation, Reach, Adoption and Effectiveness, very few addressed Maintenance/sustainability, which could reflect insufficient or lack of long-term funding for research as well as insufficient resources more broadly within Indigenous communities. More community generated and sustained funding is required. (3) Additional thoughtfulness and application of the extended RE-AIM to include a greater emphasis on equity and sustainability in D&I and treatment research among Indigenous communities was lacking. Future research could consider opportunities to expand RE-AIM to include adaptations to the domains that support the use of the framework specifically within Indigenous communities. (4) The field could benefit from a comprehensive discussion on applying Indigenous frameworks and worldviews within the context of Western D&I frameworks, models or theories. (5) CBPR plays an important role in D&I with Indigenous communities and our findings highlighted the need for more detailed descriptions of how this work is defined, employed, and assessed within partnerships. (6) Local research capacity building and front-loading engagement activities are needed to help with Reach, Adoption, and sustainment. Successful research partnerships require attention to trust and long-term relationship building. As models of sustainability evolve in the broader D&I community to reflect the challenges in the fit between interventions and local context, we hope that there will be more application of these methods and techniques to ensure interventions serve the needs of Indigenous communities.

## Author contributions

KH: Conceptualization, Data curation, Formal analysis, Funding acquisition, Methodology, Project administration, Resources, Software, Supervision, Validation, Visualization, Writing – original draft, Writing – review & editing, Investigation. OO: Conceptualization, Methodology, Writing – review & editing. MN: Conceptualization, Formal analysis, Methodology, Writing – review & editing. MR: Data curation, Formal analysis, Methodology, Software, Writing – original draft, Writing – review & editing. KB: Data curation, Formal analysis, Methodology, Software, Writing – review & editing. MB: Data curation, Formal analysis, Methodology, Writing – review & editing. JH: Conceptualization, Writing – review & editing. AH-V: Conceptualization, Writing – review & editing. AV: Conceptualization, Writing – review & editing. CP: Conceptualization, Writing – review & editing. KO: Conceptualization, Writing – review & editing. PB: Conceptualization, Writing – review & editing. KW: Writing – review & editing. LB: Writing – review & editing. MM: Writing – review & editing. KV: Conceptualization, Writing – review & editing, Data curation, Methodology. AC: Conceptualization, Writing – review & editing, Data curation, Methodology.
