# Supplementary material for: Coming together for something good: recommendations from a scoping review for dissemination and implementation science to improve indigenous substance use disorder treatment
Source: Front Public Health. 2023 Oct 17;11:1265122. doi: 10.3389/fpubh.2023.1265122 (PMC10616787; doi:10.3389/fpubh.2023.1265122)
Supplement: Supplementary file 1 [file Table_1.DOCX]

**Data extraction table: Criteria and operational definitions***

| **Section 1: Criteria relating to the key characteristics of the article** | |
| --- | --- |
|  | |
|  | |
| Lead/First author and pub year | First author of article and year of publication |
| Aim of study | Whether the author states the primary aim of the study (e.g., feasibility, acceptability, effectiveness); can list more than one |
| Study funding sources | Whether the authors list where the funding is coming from (e.g., NIH) |
| Population description | Whether the authors describe their intended racial/ethnic/Indigenous population |
| Country in which the study was conducted | List the country where the research was conducted |
| Indigenous community | Identify whether the community is AI/AN; Aboriginal (Australian); Māori or First Nations |
| Name of Indigenous Community/Tribe | If listed by the authors, identify which Tribe/community |
| Study Population Inclusion Criteria | Whether authors include the characteristics of the inclusion criteria |
| Study Population Exclusion Criteria | Whether the authors list the characteristics for the exclusion criteria  (yes/no/unclear) |
| Total Number of Participants | Sample size  (yes/no/unclear) |
| Treatment modality or intervention name/medications | Whether the authors list the evidence-based intervention being implemented (e.g., CRA; MI; CM)  (yes/no/unclear) |
| Intervention Level | Whether the authors identify whether the treatment is implemented at the individual/family/community/org/environment/society level. Can be more than one.  (yes/no/unclear) |
| Primary substance | Whether the authors discuss what substance is the intervention most trying to change/impact. Authors may identify a few, but if it’s not clear in the text look at tables to see which substance they are focusing on the most. |
| Diagnosis-Substance use disorder | Whether the authors specify if their participants/clients had any SUD diagnosis |
| Study design | Study design (i.e., **RCT** [any form of randomization]; case study [in this instance only qualitative]; mixed methods [both qual and quant with meaningful description of how each source of data was integrated; if there’s any randomization list it as an RCT]; **quantitative** [secondary data analysis, etc]; **qualitative**; **protocol/methods paper** [papers where the authors discuss their study/design usually before implementing their RCT/project etc]) |
| Cultural adaptation/centering of EBT/I | Whether the authors mention culturally adapting or centering the intervention before or during implementation |
| Formal cultural adaptation/centering/process/framework | Whether the authors mention if the adaptation/centering completed using a framework (can list CBPR here but also list others like ADAPT-IT; Two-eyed seeing; etc) |
| Implementation strategy/framework/model used | Whether the authors discuss any frameworks such as CBPR, Western implementation frameworks (e.g., EPIS, ISF)/community engaged approaches/Indigenous frameworks or worldviews (e.g., the He Pikinga Waiora Implementation Framework) |
| Contributions of Indigenous knowledge/culture to intervention implementation approach/strategy | Whether the authors provide examples around connection with land, history, language, world-view, values, sovereignty, decolonizing methodologies, or focus on kinship/relational  (yes/no/unclear) |
| **Section 2: Criteria relating to reporting at the RE-AIM dimension criteria level** | |
| **REACH** | |
| Authors discuss the intended audience and who actually participated | Whether authors report either the percentage of individuals excluded or the characteristics of those excluded (% excluded or characteristics)  Whether authors report the number, proportion, representativeness of individuals who participated in the EBT/I  (yes/no/unclear) |
| Authors discuss how we can better reach or engage the intended audience | Whether authors explicitly mention engagement strategies, impacts of SDOH/social dimensions/equity issues related to participation/lack of participation  (yes/no/unclear) |
| **EFFECTIVENESS** | |
| Was the EBT effective | Whether authors explicitly identify a primary or set of primary outcomes and for whom the outcomes were positive  (yes/no/unclear) |
| Any negative and/or unintended effects | Whether authors measure broader outcomes (e.g., quality of life) or iatrogenic effects or heterogeneity of effects  (yes/no/unclear) |
| Does the EBT continue to be effective at various time points over time | Whether the authors identify if the positive outcomes persist at end of treatment/follow-up (1-12 months post intervention/treatment phase)  (yes/no/unclear) |
| **ADOPTION** | |
| *Staff Level* | |
| Where was the EBT applied and by who | Whether authors discuss the number, proportion and representativeness of the staff/interventionists who deliver the program  Whether authors discuss which/if staff/settings continue to deliver the intervention  (yes/no/unclear) |
| *Setting Level* | |
| Which sites/staff were invited and which were excluded. Who participated and why | Whether authors report characteristics of settings participating compared to either nonparticipants or some relevant resource data  Whether authors report the percentage of settings excluded or the reasons for exclusion or both  Whether the authors discuss adaptations needed for adoption  Whether authors discuss differences in settings that adopted and applied the EBT or some that did not and why not  Whether authors discuss if there were differences in adoption by site (higher resourced v lower resourced)  (yes/no/unclear) |
| Descriptions around the setting/context and how they were supported or could be better supported to deliver the EBT | Whether the authors discuss setting level context that either facilitated or hindered implementation  (yes/no/unclear) |
| **IMPLEMENTATION** | |
|  |  |
| Was the EBT/I and or implementation strategies delivered consistently | Whether authors report consistency of implementation across staff/time/settings/subgroups (not about differential outcomes or effectiveness of the intervention, but process)  (yes/no/unclear) |
| How were the implementation strategies EBT/I adapted/culturally centered | Whether authors discuss what adaptations might be needed to promote equity and address social determinants of health  (yes/no/unclear) |
| Were costs of the implementation/delivery of the EBT discussed (cost of intervention—money) | Whether authors report cost of the intervention in terms of money  Whether authors discussed if the setting/staff have the capacity/resources to deliver the EBT/I on an ongoing basis  (yes/no/unclear) |
| Were key functions/components of the EBT delivered/discussed | Whether authors report percent of perfect delivery of the intervention (e.g., fidelity)  (yes/no/unclear) |
| Multi-level determinants addressed for implementation (e.g., internal/external/environmental factors) | Whether authors discussed factors that impacted key functions of the EBT/I delivery (e.g., importance of champion in the community) or if multi-level contextual determinants mattered for implementation (e.g., what might be needed or adapted to promote equity and address the social determinants of health)  (yes/no/unclear) |
| Discussion around how to ensure EBT *continues* to be delivered consistently with fidelity especially with reduced funding | Whether the authors describe implementation strategies on how the EBT/I could/does have long-term sustainment in the program/setting  (yes/no/unclear) |
| **MAINTENANCE/SUSTAINABILITY** | |
|  | |
| Were sustainability/implementation strategies discussed to sustain the program long-term or beyond 1 year after implementation and longer | Whether authors report if the program is still ongoing at 12-month posttreatment follow-up or later  Whether authors discuss if the health impact/benefits, outcomes and behaviours continue for patients/participants at the individual level, including patterns in health inequities overtime (beyond 1 year)  Whether authors report program activities or core components/functions of the EBT/I (& strategies) and whether those continue to be delivered at the setting/staff level w/fidelity; supports on how this has evolved and what is needed  Whether authors discuss infrastructure needed to deliver and maintain the EBT/I (e.g., partnerships, networks, coalitions, etc)  Whether authors discuss the institutionalization, capacity of staff/setting to deliver EBT/I long-term  Whether authors discuss if the population continues to be reached long-term and equitably across settings  (yes/no/unclear) |
| What multi-level contextual determinants were suggested for sustainability (informed by existing sustainability frameworks, e.g., PSAT, ISF) | Whether authors discuss the internal and external barriers and facilitators to program sustainability over time among a range of collaborators (e.g., individual level, family level, program/org level, community level, national/policy or societal levels)  (yes/no/unclear) |

*Table and definitions adapted from:

D'Lima D, Soukup T, Hull L. Evaluating the Application of the RE-AIM Planning and Evaluation Framework: An Updated Systematic Review and Exploration of Pragmatic Application. Front Public Health. 2022 Jan 26;9:755738. doi: 10.3389/fpubh.2021.755738. PMID: 35155336; PMCID: PMC8826088.

Shelton RC, Chambers DA, Glasgow RE. An Extension of RE-AIM to Enhance Sustainability: Addressing Dynamic Context and Promoting Health Equity Over Time. Front Public Health. 2020 May 12;8:134. doi: 10.3389/fpubh.2020.00134. PMID: 32478025; PMCID: PMC7235159.
