# Supplementary material for: Coming together for something good: recommendations from a scoping review for dissemination and implementation science to improve indigenous substance use disorder treatment
Source: Front Public Health. 2023 Oct 17;11:1265122. doi: 10.3389/fpubh.2023.1265122 (PMC10616787; doi:10.3389/fpubh.2023.1265122)
Supplement: Supplementary file 2 [file Table_2.DOCX]

**Search Protocol Keywords**

| **Population Keywords** | **Content Keywords** |
| --- | --- |
| American Indian and Alaska Native;  AI/AN;  Indigenous;  First Nations;  Aboriginal;  Maori; and  Native Hawaiian | Dissemination and implementation science Indigenous dissemination and implementation science;  Consolidated Framework for Implementation Research;  Explore Preparation Implementation Sustainment;  Quality Implementation Framework;  Community Based Participatory Research;  Community engaged;  Implementation strategy;  (a) intervention (b) program Adoption;  (a) intervention (b) program Adaption;  Community engagement;  Participatory action research;  two-eyed seeing/Etuapmunk;  AI/AN Western Knowledge implementation and dissemination  AI/AN Indigenous knowledge implementation and dissemination  Indigenous Implementation Framework;  Kaupapa Māori;  4R-4D Framework implementation;  Health equity implementation framework;  Constellations model implementation;  Cultural interface implementation;  Ethical space implementation;  Indigenous cultural responsiveness theory (ICRT) implementation;  Insurgent research implementation;  Expansive learning;  Hybridity;  Indigenous Métissage;  Möbius strip metaphor;  Reach Effectiveness-Adoption Implementation Maintenance;  Polycentric global epistemology implementation;  Rhizome implementation  Guswentah, or Two-row wampum implementation;  Working the hyphen AI/AN indigenous implementation;  Living on the ground implementation; and  Intersectionality |
